# The distance distribution of human microRNAs in MirGeneDB database

**DOI:** 10.1038/s41598-022-22253-6

**Published:** 2022-10-21

**Authors:** Hsiuying Wang

**Affiliations:** Institute of Statistics, National Yang Ming Chiao Tung University, Hsinchu, 30010 Taiwan

**Keywords:** Computational biology and bioinformatics, Genetics, Biomarkers, Diseases

## Abstract

MicroRNAs (miRNAs) are small single-stranded non-coding RNAs around 22 nucleotide lengths found in organisms, playing an important role in cell differentiation, development, gene regulation, and apoptosis. The distance of disease miRNA biomarkers has been used to explore the association between various diseases as well as the association between virus and disease in the literature. To date, there have been no studies on deriving the distribution of the pairwise distance of human miRNAs. As the pairwise distance of miRNA biomarkers might be a useful tool in studying the disease association, in this paper, the distance distributions of human miRNAs were derived such that they could be used to measure the closeness between miRNAs. Two distance models were used to calculate the pairwise distances of 567 Homo sapiens miRNA genes accessed from the MirGeneDB database. These miRNA pairwise distances were fitted by the normal distribution, gamma distribution, empirical cumulative distribution, and the kernel density estimation method. This is the first study to provide the distance distribution of human miRNAs. The similarity of miRNA biomarkers for several diseases was examined using the derived distributions.

## Introduction

MicroRNAs (miRNAs) are non-coding RNAs about 21–24 nucleotides long that play an important role in cell differentiation, development, apoptosis, and cell cycle regulation^1,2^. The first miRNA was discovered in the 1990s when the nematode Caenorhabditis elegans-related gene lin-14 was studied^3^. miRNA can regulate up to 30% of protein-coding genes in the human genome^4^. They are involved in the initiation and progression of many diseases, especially cancers. They can act as tumor suppressor genes or oncogenes, and they can also be regulated by tumor suppressor genes and oncogenes^5,6^. The biogenesis of miRNA can be classified into canonical and non-canonical pathways^7^. In the canonical pathway, a primary miRNA transcript is cleaved by the endoRNase Drosha to excise the precursor miRNA. The cytoplasmic RNase III Dicer cut the precursor miRNA to process into mature miRNAs. For the non-canonical miRNA biogenesis pathways, different combinations of the proteins related to the canonical pathway are involved in the non-canonical pathways.

miRNAs participate in many pathological processes and play an important role in the progression of cancers. They were very useful biomarkers for various cancers^8^. miR-613, a new-found miRNA, was involved in the development of colorectal cancer, hepatocellular carcinoma, gastric cancer, non-small cell lung cancer, and breast cancer^9^. miRNAs were studied to contribute to the development and progression of human papilloma virus-induced malignancies^10^. miR-149 played a key role in the pathogenesis of digestive system cancers including colorectal cancer, hepatocellular cancer, gastric cancer, oral cancer, pancreatic cancer, and esophageal cancer^11^. miR-34 played a considerable role in repressing tumor progression that acted as a negative regulatory factor of tumor-associated epithelial-mesenchymal transition^12^. miR-142 was involved in cellular migration, proliferation, and apoptosis in different human cancers including lung cancer, breast cancer, gynecological malignancies, cervical cancer, ovarian cancer, colon cancer, and colorectal cancer^13^. In addition to cancer, miRNAs also contributed to many other diseases including metabolic disease, mental disease, neurological diseases, and the coronavirus disease 2019 (COVID-19)^14–18^.

Another application for miRNA is to explore the association between diseases. miRNA biomarkers were used to explore the association between major depression and other diseases such as multiple sclerosis, gastroesophageal reflux, and migraine^19,20^. They were also used to explore the relationship between diabetes mellitus and colorectal cancer as well as the relationship between diabetes mellitus and Parkinson's disease^1,21^. In addition, miRNA biomarkers could be used to analyze the relationship between vaccines and adverse events^22–24^.

According to these previous studies, the distance between miRNA biomarkers has been used to explore the association between diseases. In light of this, to have a more depth study on this topic, this paper focuses on two issues. The first one is to discuss the feasibility of using the distance of miRNA biomarkers to explore disease associations; the other is to find miRNA pairwise distance distributions such that they can be used to measure the closeness between miRNAs.

To explore the first issue, phylogenetic analysis was used to study the relationship between miRNA biomarkers for different diseases or vaccines, as seen from previous related association studies^15,25^. The phylogenetic analysis was a useful tool in comparative genomics^26,27^. Phylogenetic trees of the miRNA biomarkers can be plotted to cluster the miRNA biomarkers. In a cancer miRNA biomarker study, combining the phylogenetic tree approach with a microarray method could increase the accuracy of miRNA biomarker prediction compared with the method only using the microarray analysis^27^. The result showed that many high-confidence miRNA biomarkers for particular cancers were in the same clade of a phylogenetic tree. This means that the miRNA biomarkers of the same cancer may have a smaller mean distance compared to those for different cancers. Therefore, this result motivates this study to investigate whether the miRNA biomarkers for a disease or common miRNA biomarkers of diseases also have a smaller mean pairwise distance than the overall mean distance of miRNAs. Several diseases are used to explore this issue in this study.

Since the pairwise distance of miRNAs was used as a tool in the literature, a distance threshold should be set to evaluate whether a distance value is small or not. This motivates the second research issue to find the distribution of the distance value such that a distance threshold can be calculated from the distribution function. To derive the distribution function for the distance data, first, we need to calculate the pairwise distances of all miRNAs, and then find statistical models to fit these data. Different nucleotide substitution models have been proposed to calculate the pairwise distance of gene sequences in the literature^28^. Two commonly used nucleotide substitution models, Jukes and Cantor’s (JC) one-parameter model and Kimura two-parameter model, are considered in this study to calculate the pairwise distances of miRNAs^29,30^. Several methods in deriving the distribution functions for the distances based on these nucleotide substitution models are compared in this study.

## Materials and methods

MirGeneDB is a miRNA database. The version MirGeneDB 2.1 is available at https://mirgenedb.org/. The miRNA genes stored in this database have been validated and annotated^31,32^. The MirGeneDB stores miRNA gene entries from 75 metazoan species including 567 human miRNA genes. miRNA precursor sequences, mature sequences, and others can be accessed from this database. The mature miRNA is the functional one that can target mRNAs to regulate their expression. Therefore, the pairwise distances of mature miRNAs are used to measure the similarity of miRNAs in this study.

### Distance model

The JC one-parameter model and the Kimura two-parameter model were reviewed in this subsection. The JC one-parameter model is a frequently used model assuming that substitutions occur with equal probability among the four nucleotide types, A, T, C, and G. Let $K$ denote the number of substitutions per site since the time of divergence between two sequences with length $L$. Let $X$ denote the number of different sites between these two sequences. Under the JC one-parameter model, we have

1 $$K_{1} = - \frac{3}{4}\ln \left( {1 - \frac{4}{3}\hat{p}} \right)$$

where $\hat{p} = X/L$ is the observed proportion of different nucleotides between two sequences. The value $K_{1}$ is used as the first distance of two miRNA sequences in this study. An approximated estimator for the sampling variance of $K_{1}$ is^33,34^

$$V(K_{1} ) = \frac{{\hat{p} - \hat{p}^{2} }}{{L\left( {1 - \frac{4}{3}\hat{p}} \right)^{2} }}$$

Another frequently used model is the Kimura two-parameter model^30^. Let $\hat{P} = X_{1} /L$ and $\hat{Q} = X_{2} /L$ be the observed proportions of transitional and transversional differences between two sequences, respectively, where $X_{1}$ and $X_{2}$ denote the numbers of transitional and transversional differences between the two sequences. Then the number of nucleotide substitutions per site between the two sequences, $K_{2}$, is estimated by

2 $$K_{2} = \frac{1}{2}\ln \left( {\frac{1}{{1 - 2\hat{P} - \hat{Q}}}} \right) + \frac{1}{4}\ln \left( {\frac{1}{{1 - 2\hat{Q}}}} \right)$$

The value $K_{2}$ is used as the second distance of two miRNA sequences in this study.

### Method

The pairwise distances of the 567 miRNAs were calculated using the two nucleotide substitution models, respectively. To find a statistical distribution to fit these distance data, we use the two-sample Kolmogorov–Smirnov test to evaluate the derived distributions. The normal distribution, gamma distribution, empirical cumulative distribution, and the kernel density estimation method were used to fit the distance data. The empirical cumulative distribution and kernel density estimation are reviewed as follows.

Let $F_{{}} (x)$ be the cumulative distribution of the pairwise distance of mature miRNA sequences. We use the calculated distance data to estimate $F_{{}} (x)$. Let $\hat{F}_{n} (x)$ be the empirical cumulative distribution based on $n$ distance data, $x_{1} , \ldots ,x_{n}$. The definition of $\hat{F}_{n} (x)$ is

3 $$\hat{F}_{n} (x) = \frac{1}{n}\sum\limits_{i = 1}^{n} {I_{{(x_{i} \le x)}} (x} )$$

where $I_{A} (x)$ denotes the indicator function that $I_{A} (x) = 1$ when $x \in A$ and $I_{A} (x) = 0$ otherwise. $\hat{F}_{n} (x)$ can be used to estimate $F_{{}} (x)$.

Another methodology is the kernel density estimation method. Unlike the empirical cumulative distribution method, the kernel density estimation method is to estimate the density function instead of the cumulative distribution. The estimated density is

$$\hat{f}_{h} (x) = \frac{1}{n}\sum\limits_{i = 1}^{n} {Kernel_{h} } (x - x_{i} ) = \frac{1}{nh}\sum\limits_{i = 1}^{n} {Kernel\left( {\frac{{x - x_{i} }}{h}} \right)}$$

where $Kernel$ is the kernel function, a non-negative function, and $h > 0$ is a smoothing parameter called the bandwidth^35^.

## Results

### The JC model

**Figure 1:**
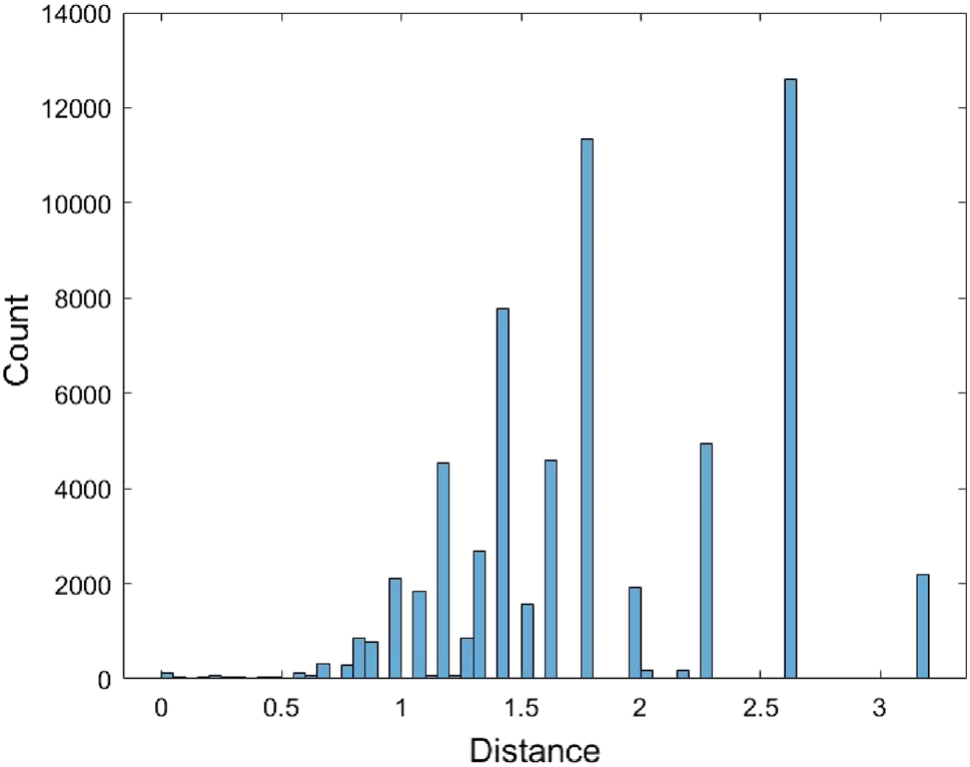
The histogram of the JC model distance data.

The pairwise distances between the 567 human mature miRNA sequences were calculated using the JC model by MEGA software^36^ (version MEGA 11, https://www.megasoftware.net/). These miRNA sequences were first aligned and then the distances were calculated. For the distance calculation using the MEGA software, there are several options for dealing with the gaps. The gaps/missing data treatment option was selected to be the pairwise deletion. Some distances could not be calculated and were returned as n/c in MEGA. It is noted that the formula of the JC model distance (1) requires one condition $1 - \frac{4}{3}\hat{p} > 0$, otherwise, the distance value cannot be calculated. There are a total of 62,435 calculated pairwise distances for these miRNAs (Supplementary S1). The range of these distances is (0, 3.1756) and the average of the 62,435 distances is 1.8156. To find a distribution to fit these 62,435 distances, we first plot the histogram of these distances (Fig. 1).

**Figure 2:**
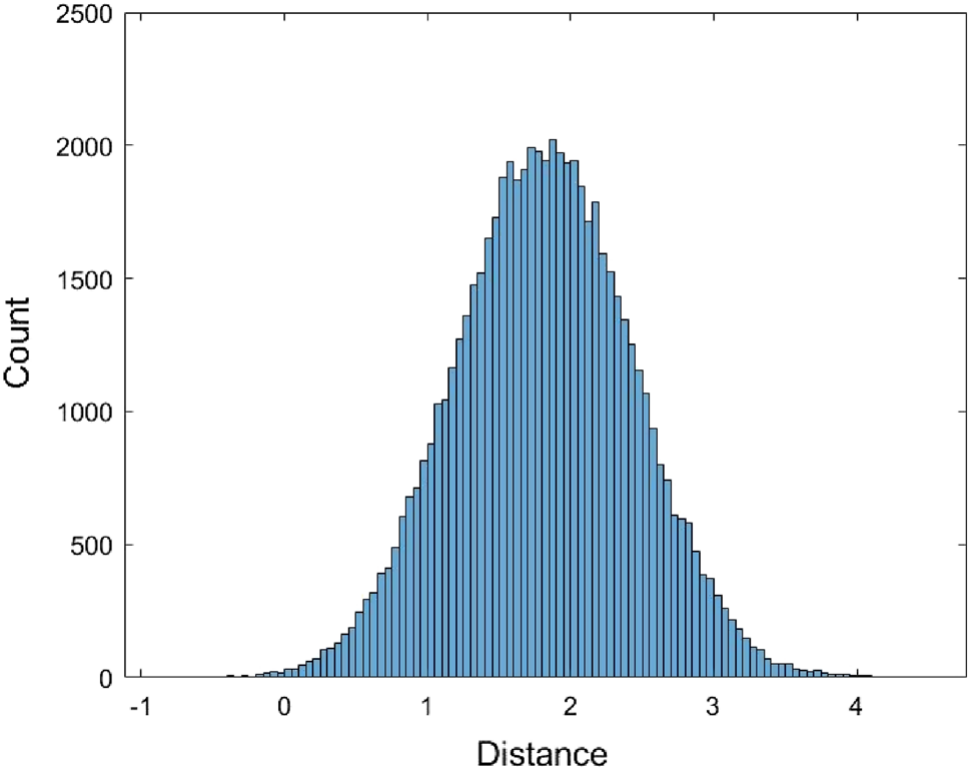
The histogram of 62,435 data generated from the fitted normal distribution of the JC model distance data.

**Figure 3:**
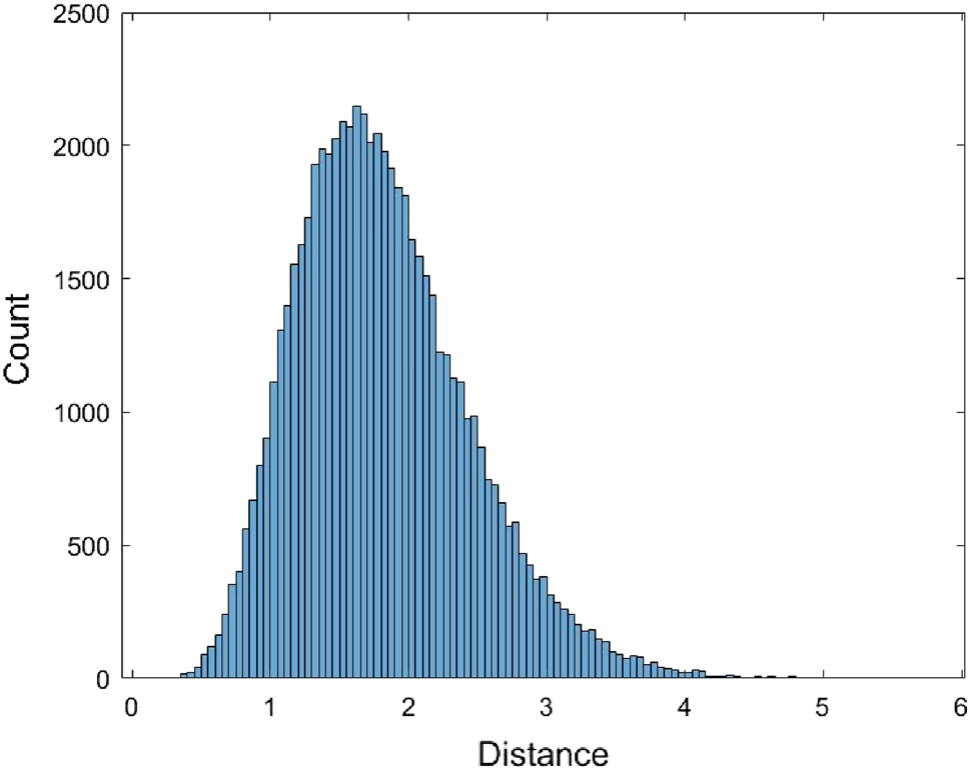
The histogram of 62,435 data generated from the fitted Gamma distribution of the JC model distance data.

The histogram shows that the data is skewed. Therefore, it is not suitable to use a symmetrical distribution to fit the data such as the normal distribution. Nevertheless, the normal distribution $N(\mu ,\sigma^{2} )$ with mean $\mu$ and variance $\sigma^{2}$ was used to fit the data. Another non-symmetrical distribution Gamma distribution $Gamma(\alpha ,\beta )$ with shape parameter $\alpha$ and scale parameter $\beta$ was used to fit the data. When using the normal distribution to fit the data, the estimated value of $\mu$ is $\hat{\mu } = {1}{\text{.81558}}$ and the estimated value of variance $\sigma$ is $\hat{\sigma } = {0}{\text{.6218}}$. When fitting the data with the Gamma distribution, the estimated value for $\alpha$ and $\beta$ are $\hat{\alpha } = 8.5257$ and $\hat{\beta } = 0.212954$, respectively. Figures 2 and 3 are the histograms of 62,435 data generated from the fitted normal distribution and Gamma distribution, respectively.

**Figure 4:**
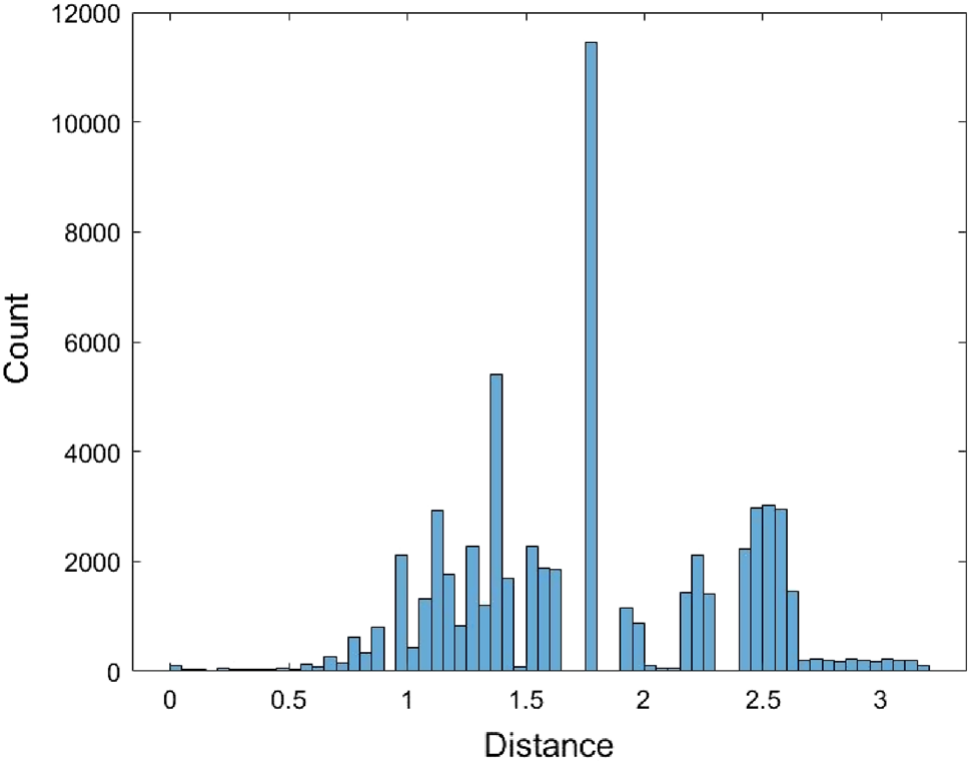
The histogram of 62,435 data generated from the fitted empirical distribution of the JC model distance data.

Next, the empirical cumulative distribution was used to fit the data. The piecewise linear approximation method is used to smooth the distribution. The empirical cumulative distribution was obtained by the Matlab software version R2019b (https://www.mathworks.com/products/matlab.html). Figure 4 shows the histogram of 62,435 data generated from the fitted empirical cumulative distribution.

**Figure 5:**
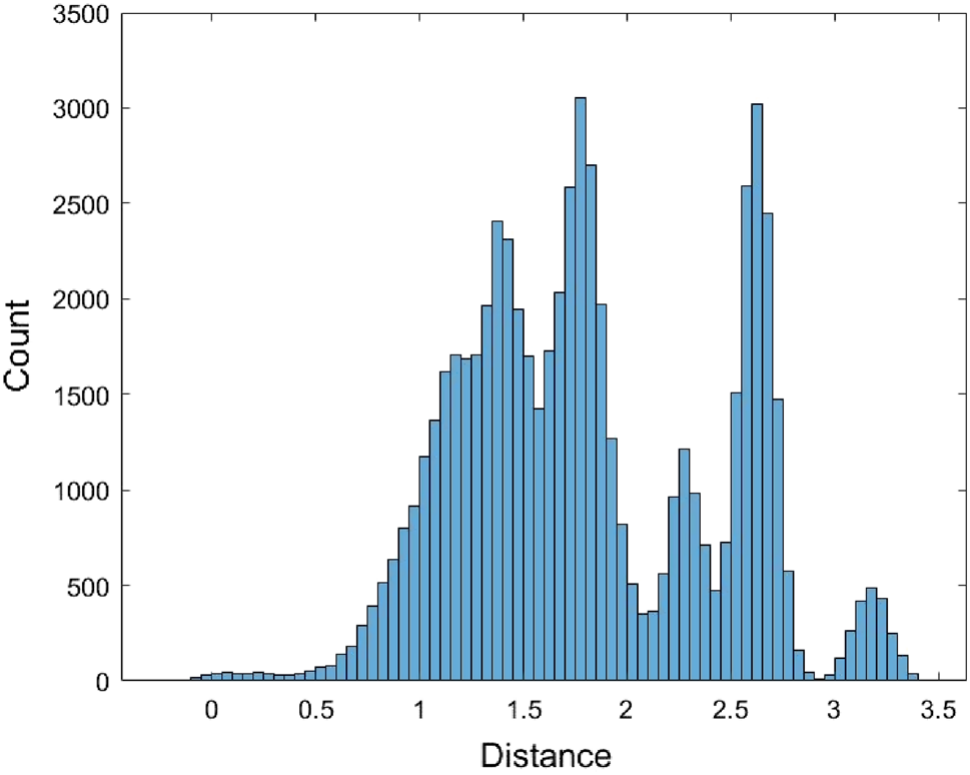
The histogram of 62,435 data generated from the fitted kernel density estimation method of the JC model distance data.

Finally, the kernel density estimation method was used to fit the data. The kernel function used in this method is the normal distribution. Figure 5 shows the histogram of 62,435 data generated from the kernel density estimation method. In Fig. 5, the bandwidth in the kernel density estimation method is set to 0.0836826 in the Matlab software.

The two-sample Kolmogorov–Smirnov (KS) test was used to evaluate the distribution fitting results. The KS test can be used to test the similarity of two distributions. If the sample size is large, the KS test will lead to a rejection result unless the two distributions are almost the same. Therefore, a moderate size sample was used to perform the KS test. Here 70 data were generated from each of the four fitted distributions, and the p-values of the KS test were calculated. The fitting process and the KS test were performed 500 times for each method and the average of the p-values are provided in Table 1.

**Table 1:** The average p-values of the Kolmogorov–Smirnov test for the JC model distance data.

Model	Estimated parameter (one time)	p-value
Normal distribution	$\hat{\mu } = {1}{\text{.81558}}$ $\hat{\sigma } = {0}{\text{.6218}}$	0.0455
Gamma distribution	$\hat{\alpha } = 8.5257$ $\hat{\beta } = 0.212954$	0.0772*
Empirical cumulative distribution	Piecewise linear approximation	0.0069
Kernel density estimation	Kernel = normal distribution Bandwidth = 0.0836826 Support = unbounded	0.1114*

*The p-values greater than 0.05 are denoted as asterisk.

From Table 1, we can see that only the Gamma distribution and the kernel density estimation method have an average of p-values greater than 0.05. It concludes that the fitted Gamma distribution and the kernel density estimation method are preferable to the normal distribution and the empirical cumulative distribution. The kernel density estimation method with the highest p-value is preferred. Since the kernel density estimation method can fit these data better than the other methods, the percentiles based on this method were calculated and tabulated in Table 2. The qth percentile denotes the number for which q% of the data falls below this number. For example, the 25^th^ percentile indicates the point where 25% of the data is less than this number. The Matlab codes for performing the distribution fitting methods and the KS test for the JC model distance data are provided as supplementary materials (Supplementary Matlab code 1).

**Table 2:** The percentiles of JC model distance based on the kernel density estimation method.

q	The qth percentile	q	The qth percentile
5	0.9178	55	1.7922
10	1.0642	60	1.8483
15	1.1656	65	1.9400
20	1.2577	70	2.2190
25	1.3420	75	2.3635
30	1.4093	80	2.5454
35	1.4802	85	2.6083
40	1.5753	90	2.6619
45	1.6702	95	2.7441
50	1.7384	100	3.4342

### The Kimura model

**Figure 6:**
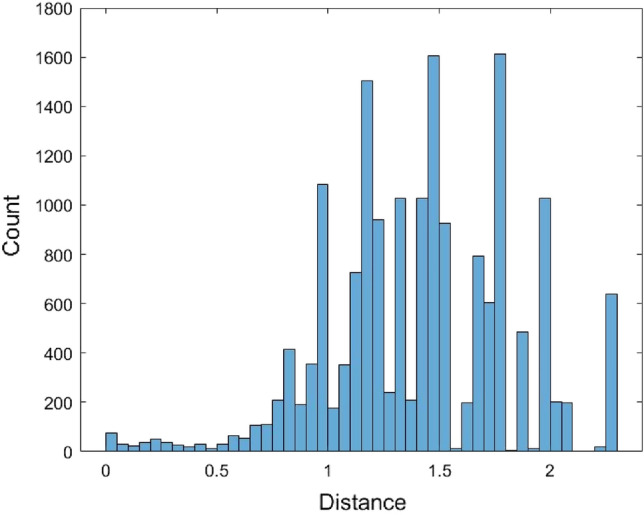
The histogram of 17,519 Kimura model distance data.

The Kimura model was also used as the distance model to calculate the pairwise distances of the 567 human mature miRNA sequences. These miRNA sequences were first aligned. As in the JC model case, some distances could not be calculated and were returned as n/c in MEGA. There are a total of 17,519 calculated pairwise distances for these miRNAs based on the Kimura model (Supplementary S2). It is noted that the formula of the Kimura model distance (2) requires two conditions, $\frac{1}{{1 - 2\hat{P} - \hat{Q}}} > 0$ and $\frac{1}{{1 - 2\hat{Q}}} > 0$, otherwise, the distance value cannot be calculated. These conditions are more restricted than the JC model, and this might lead to fewer calculated pairwise distances calculated by the Kimura model than by the JC model. The range of these 17,519 distances is (0, 2.2834). Figure 6 is the histogram of these distance data.

**Figure 7:**
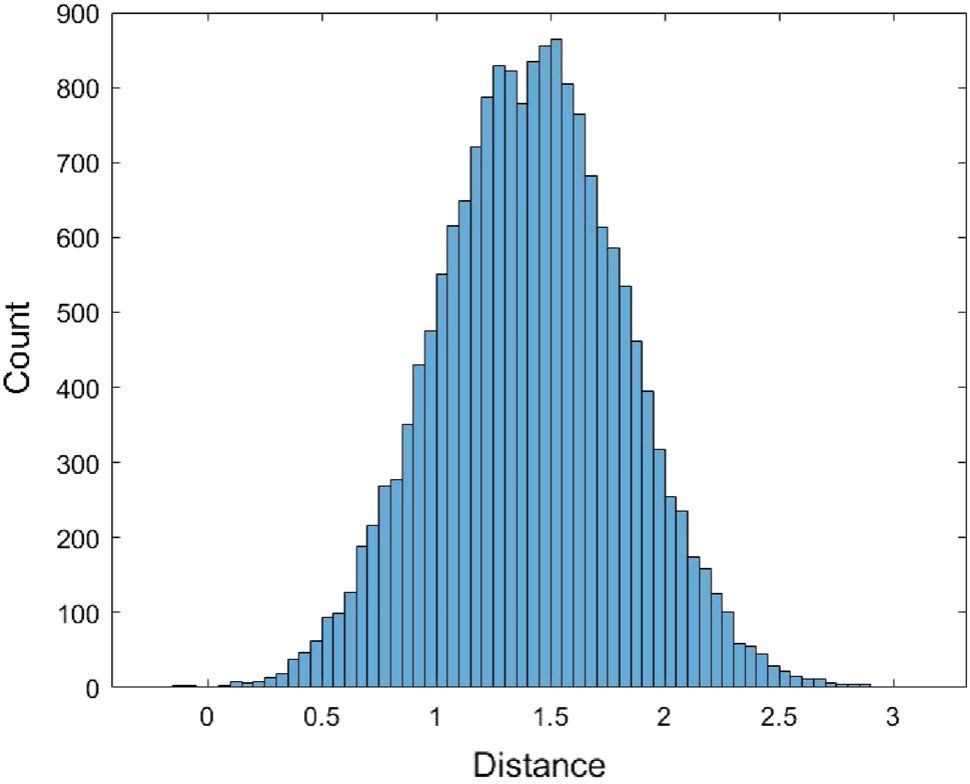
The histogram of 17,519 data generated from the fitted normal distribution of the Kimura model distance data.

**Figure 8:**
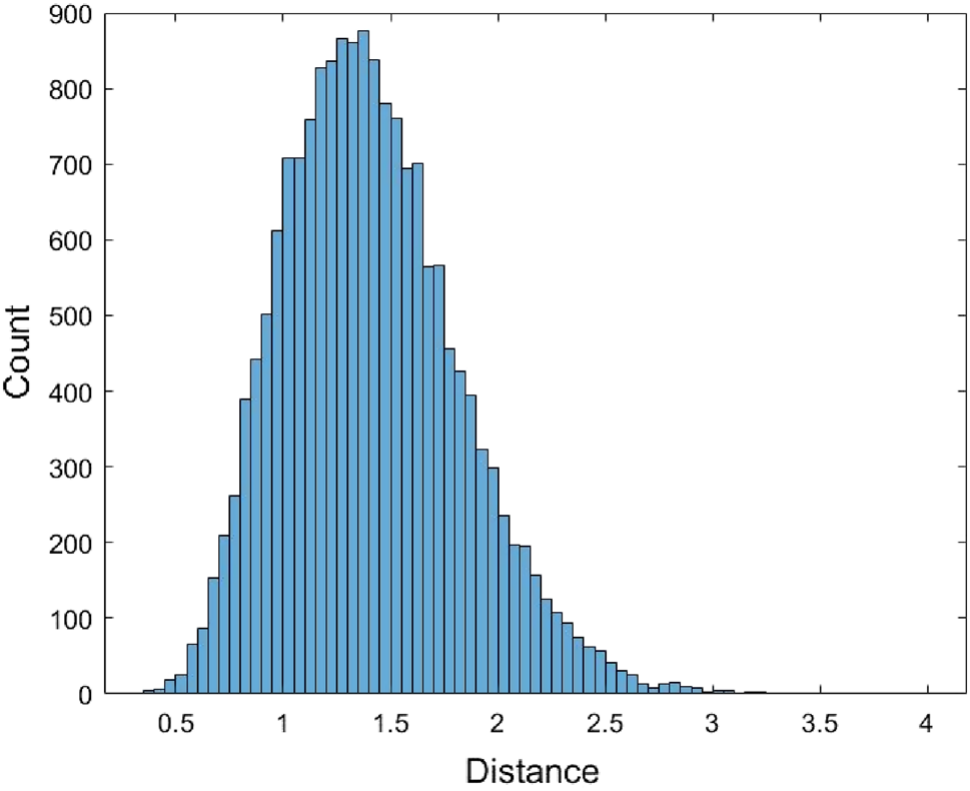
The histogram of 17,519 data generated from the fitted Gamma distribution of the Kimura model distance data.

**Figure 9:**
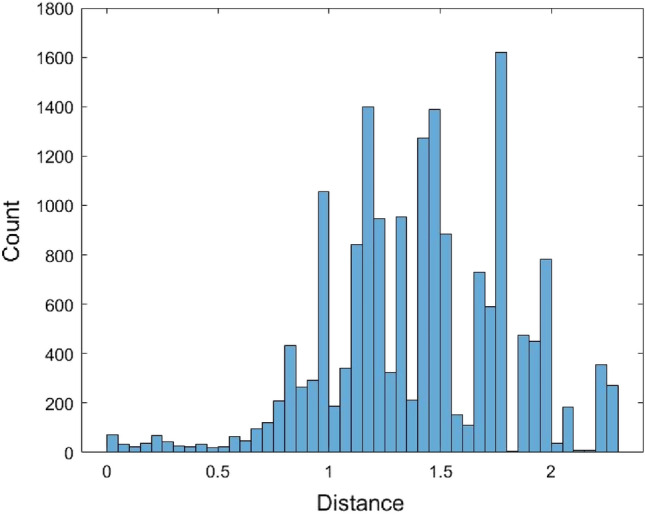
The histogram of 17,519 data generated from the fitted empirical distribution of the Kimura model distance data.

**Figure 10:**
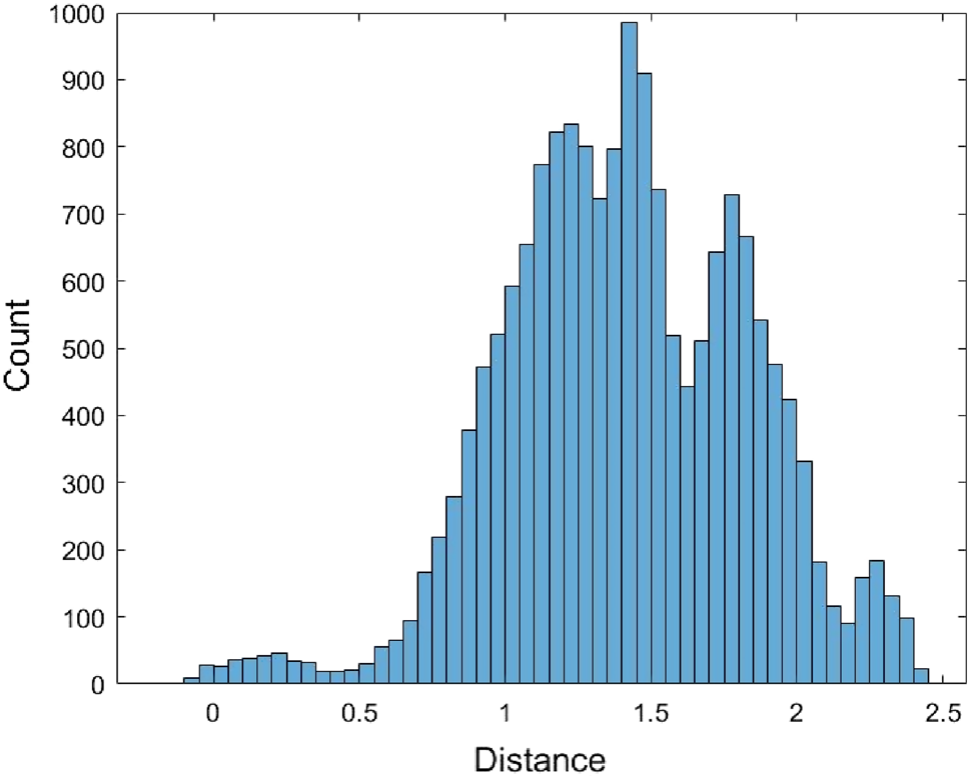
The histogram of 17,519 data generated from the fitted kernel density estimation method of the Kimura model distance data.

The normal and the Gamma distributions were used to fit the Kimura model distance data as well as the empirical method and the kernel density estimation method. The histograms of 17,519 data generated from these four fitted distributions are provided in Figs. 7, 8, 9, and 10.

As in the JC model case, 70 data generated from each of the four fitted distributions for the Kimura model distance data were used to perform the KS test. The model fitting process and the KS test were performed 500 times for each method and the average of the p-values are provided in Table 3.

**Table 3:** The average p-values of the Kolmogorov–Smirnov test for the Kimura model distance data.

Model	Estimated parameter (one time)	p-value
Normal distribution	$\hat{\mu } = {1}{\text{.41315}}$ $\hat{\sigma } = {0}{\text{.414215}}$	0.3104
Gamma distribution	$\hat{\alpha } = {11}{\text{.6393}}$ $\hat{\beta } = {0}{\text{.121412}}$	0.3002
Empirical cumulative distribution	Piecewise linear approximation	0.3137
Kernel density estimation	Kernel = normal distribution Bandwidth = 0.0693462 Support = unbounded	0.3457

In Table 3, the average p-values of the four fitted distributions are all greater than 0.05. The kernel density estimation method has the highest average p-value. It indicates the kernel density estimation method is most preferable. As a result, the distribution derived by this method can be an approximate distribution of the Kimura model distance data. The quantiles of this method are tabulated in Table 4. The Matlab codes for performing the distribution fitting methods and the KS test for the Kimura model distance data are provided as supplementary materials (Supplementary Matlab code 2).

**Table 4:** The percentiles of Kimura model distance based on the kernel density estimation method.

q	The qth percentile	q	The qth percentile
5	0.7737	55	1.4569
10	0.9161	60	1.5043
15	1.0043	65	1.5649
20	1.0785	70	1.6592
25	1.1431	75	1.7363
30	1.1948	80	1.8011
35	1.2497	85	1.8667
40	1.3051	90	1.9497
45	1.3612	95	2.0717
50	1.4100	100	2.4966

### Applications

In this section, the disease miRNA biomarkers from four papers were used to investigate the similarity of biomarkers^19,23,37,38^. The distributions of miRNA pairwise distances derived from the kernel density estimation method were used to examine whether these miRNA biomarkers are relatively similar compared with all miRNAs.

First, the association between anti-NMDA receptor encephalitis and vaccination is discussed. Anti-NMDA receptor encephalitis is an acute autoimmune disorder that occurs more often in females than in males^38,39^. The cause of this disease is usually unknown. Tumors or vaccination might trigger this disease. Vaccination against H1N1 influenza, tetanus, diphtheria, pertussis, poliomyelitis, Japanese encephalitis, and COVID-19 were reported to be related to anti-NMDA receptor encephalitis^15,22,23^. Since several anti-NMDA receptor encephalitis cases have been reported to be triggered by vaccination, the miRNA biomarkers of anti-NMDA receptor encephalitis and the miRNA biomarkers of these vaccine-related viruses or bacteria may be also correlated. Thus, these miRNA biomarkers have been used to explore their association. The 25 miRNAs listed in Table 5 were used to explore the association between anti-NMDA receptor encephalitis and vaccination^23^. Among the 25 biomarkers, the biomarkers of anti-NMDA receptor encephalitis were let-7a, let-7b, let-7d, and let-7f. Some of these four are also biomarkers for the H1N1 vaccine. One of these 25 miRNAs that is underlined in Table 5 is not in the MirGeneDB database. The details of these miRNA biomarkers are provided in Supplementary S3. The means of the JC model distance and the Kimura model distance for these biomarkers in the MirGeneDB are 1.59589 and 1.05947, respectively. These means 1.59589 and 1.05947 are the 40.64th and 18.85th percentiles of the fitted kernel density estimation distribution for the JC model and the Kimura model, respectively. It is noted that the mean values of all pairwise distance data for the JC model and the Kimura model are 1.8156 and 1.4132, respectively, which are 57.10th and 50.78th percentiles of the corresponding kernel density estimation distributions. Compared with the 57.10th and 50.78th percentiles, the 40.64th and 18.85th percentiles of these miRNA biomarkers are relatively small. By applying the Wilcoxon rank sum test, the distance values of these biomarkers are significantly different from all pairwise distances with a p-value of 2.1419e−04 for the JC model and with a p-value of 7.8110e−05 for the Kimura model. It indicates that these miRNA biomarkers have a smaller mean distance compared with the overall mean of all pairwise distances of miRNAs.

**Table 5:** The pairwise distance of the miRNA biomarkers of anti-NMDA receptor encephalitis and vaccination.

miRNA biomarkers (the miRNA with the underline mark was not found in MirGeneDB)	miR-323, miR-491, miR-654, miR-10a, miR-31,miR-29a, miR-148a, miR-146a, miR-202, miR-342, miR-206, miR-487b, miR-576, miR-555, miR-145, miR-101, miR-19b, miR-33a, miR-155, miR-29b, let-7a, let-7b, let-7c,let-7d, let-7f
The mean of the JC model distance (qth percentile)	1.59589 (40.64th percentile)
The mean of the Kimura model distance (qth percentile)	1.05947 (18.85th percentile)

In addition to vaccination, tumors might trigger anti-NMDA receptor encephalitis^40–42^. Ovarian teratoma, dura mater lesions, neuroendocrine tumor, mediastinal teratoma, testis teratoma, and small-cell lung cancer were associated with anti-NMDA receptor encephalitis^37^. The 27 miRNAs listed in Table 6 were used to explore the association between anti-NMDA receptor encephalitis and tumors^37^. Among these 27 biomarkers, some of the four anti-NMDA receptor encephalitis biomarkers let-7a, let-7b, let-7d, and let-7f are also associated with ovarian teratomas, neuroendocrine tumors, testis teratomas, and small-cell lung cancer^37^.

**Table 6:** The pairwise distance of the miRNA biomarkers of anti-NMDA receptor encephalitis and tumors.

miRNA biomarkers (the miRNAs with the underline mark were not found in MirGeneDB.)	miR-371, miR-372, miR-373, miR-129, miR-103, miR-107, miR-29b, miR-19a, miR-142, miR-26b, miR-421, miR-934, miR-22, miR-34a, miR-214, miR-196a, miR-629, miR-555, miR-657, miR-27a let-7b, let-7f, let-7a, let-7d, miR-492, miR-150, miR-620
The mean of the JC model distance (qth percentile)	1.71356 (47.80th percentile)
The mean of the Kimura model distance (qth percentile)	0.99470 (14.55th percentile)

Four of these 27 miRNAs that are underlined in Table 6 are not in the MirGeneDB database. The details of these miRNA biomarkers are provided in Supplementary S4. The means of these biomarkers in MirGeneDB are 1.71356 and 0.99470 for the JC model and the Kimura model, respectively. These means are the 47.80th and 14.55th percentiles of the kernel density estimation distribution for the JC model and the Kimura model, respectively. For the JC model, the 47.80th percentile is not sufficient to indicate that these miRNA biomarkers are highly similar. For the Kimura model distance, the 14.55th percentile indicates that they are highly similar. By applying the Wilcoxon rank sum test, the JC model distances of these biomarkers are not significantly different from all pairwise JC model distances with a p-value of 0.3906, but the Kimura model distances of these biomarkers are significantly different from all pairwise Kimura model distances with a p-value of 1.1732e−06. This result indicates that these miRNA biomarkers have a relatively high similarity by considering the Kimura model.

In the third case, the miRNAs used to link migraine and major depression are considered. Chen and Wang explored the association between major depression and migraine based on 12 miRNA biomarkers listed in Table 7^19^. Among the 12 miRNA biomarkers that could be identified to be associated with migraine from the literature, 11 of them were related to major depression^19^. It might indicate an association between migraine and major depression. The details of these miRNA biomarkers are provided in Supplementary S5. The means of the JC model distance and the Kimura model distance for these miRNAs in MirGeneDB are 1.88649 and 1.14269, respectively. These means are in the 62.60th and 25.57th percentiles of the kernel density estimation distribution for the JC model and the Kimura model distance data, respectively. For the JC model distance, the 62.60th percentile indicates that these miRNA biomarkers are less similar than the overall miRNAs, while for the Kimura model case, the 25.57th percentile still shows a high similarity. By applying the Wilcoxon rank sum test, the JC model distances of these biomarkers are not significantly different from all pairwise JC model distances with a p-value of 0.2309, but the Kimura model distances of these biomarkers are significantly different from all pairwise Kimura model distances.

**Table 7:** The pairwise distance of 12 miRNA biomarkers of major depression and migraine.

miRNA biomarkers	miR-590, miR-34a, miR-382, miR-30a, miR-375, miR-27a, miR-181a, let-7b, miR-22, miR-155, miR-126, let-7g
Average of JC model distance (qth percentile)	1.88649 (62.60th percentile)
Average of Kimura model distance (qth percentile)	1.14269 (25.57th percentile)

Finally, the miRNAs related to apoptosis of colorectal cancer are considered. The 40 miRNAs listed in Table 8 have been studied to mediate the apoptosis pathway associated with colorectal cancer^38^. One of these 27 miRNAs that are underlined in Table 6 is not in the MirGeneDB database. The details of these miRNA biomarkers are provided in Supplementary S6. The means of the pairwise JC model and Kimura model distance of these biomarkers in the MirGeneDB database are 1.651 and 1.314, respectively. These means are the 43.54th and 41.38th percentiles of the kernel density estimation distribution of the JC model and the Kimura model distance, respectively. Although these miRNA biomarkers do not have very high similarity, they have a higher similarity than average. By applying the Wilcoxon rank sum test, the JC model distances of these biomarkers are significantly different from all pairwise JC model distances with a p-value of 9.0868e−05. For the Kimura model, the p-value of the Wilcoxon rank sum test is 0.0530. If the cutoff point 0.05 is used for the p-value, the distances of these biomarkers are not significantly different from all pairwise Kimura model distances. If a slightly relaxed p-value criterion is considered, it can be said that the distances for these biomarkers are different from all pairwise Kimura model distances.

**Table 8:** The pairwise distance of miRNAs related to the apoptosis of colorectal cancer.

miRNA biomarkers (the miRNAs with the underline mark were not found in MirGeneDB)	miR-92a, miR-766, miR-21,miR-96, miR-17, miR-100, miR-365, miR-378, miR-18a, miR-125a, miR-125b, miR-10b, miR-200c, miR-217, miR-206, miR-210, miR-23a, miR-520g, miR-129, miR‐32, miR-218, miR-195, miR-491, miR-7, miR-148a, miR-708, miR-182, miR-34a, miR-133b, miR-145, miR-143, miR-342, miR-26b, miR-630, miR-135b, miR-196b, miR-22, miR-532, miR-769, miR-20a
The mean of the JC model distance (qth percentile)	1.88649 (43.54th percentile)
Average of Kimura model distance (qth percentile)	1.14269 (41.38th percentile)

From these analyses, three of the four cases show that the common miRNA biomarkers of two diseases or the miRNA biomarkers of a disease have a smaller distance mean compared with the overall mean distance for one or two distance models. Only one case does not have this phenomenon. Compared with the other three cases, this case only has 12 biomarkers. It is not clear whether a lower number of biomarkers would lead to different outcomes than the other three cases.

It is known that the miRNA seed played a more important role in target recognition than the rest of the miRNA sequence^43^. The use of miRNA seed sequences for biomarker analysis is also an interesting topic that could be a future study. In addition, the mean distances of miRNAs in the same seed family for several cases are examined using the derived distributions. Table 9 provides the mean distances for the JC model and Kimura model of three seed families. In these three cases, all of them have a smaller mean distance than the overall mean. The percentiles of these means are very small. All of them are smaller than one percentile. It shows the very high similarity of the miRNA mature sequences in each of the three families. It is very likely that the mature sequences in other seed families also have high similarities. In addition, the KS test was also used to test the similarity between the distribution derived by the kernel density estimation method for all pairwise distances and that for the pairwise distances of the LET-7 family. The distributions are significantly different with p-values 2.3048e−31 and 1.7902e−30 for JC and Kimura model distance, respectively.

**Table 9:** The distance of miRNAs in three seed families.

Family	Seed	Number	JC average distance (percentile)	Kimura average distance (percentile)
LET-7	GAGGUAG	12	0.1190 (0.24th percentile)	0.1206 (0.66th percentile)
MIR-1	GGAAUGU	3	0.1388 (0.27th percentile)	0.1435 (0.76th percentile)
MIR-7	GGAAGAC	3	0 (0)	0 (0)

## Conclusion

miRNAs have been widely used as disease biomarkers for various diseases. The association between diseases has been explored by analyzing their miRNA biomarkers. As miRNAs are involved in disease mechanisms, there might be an association between two diseases if these two diseases have many common miRNA biomarkers or have miRNA biomarkers with high similarity. The pairwise distance distribution of miRNAs can be used to assess the proximity between miRNAs. To the best of my knowledge, there have been no studies exploring the distribution of miRNA pairwise distance. To this end, the approximate distributions of miRNA pairwise distances based on the JC and Kimura substitution models were derived in this study. Using these derived distributions, the similarity of miRNA biomarkers for several diseases was evaluated. The results show that the mean distances of the miRNA biomarkers are smaller than the overall mean disease in three of the four studied cases for some distance model. In conclusion, this paper provides approximate distributions of miRNA pairwise distance that can be used to measure the similarity of miRNAs, and to study the similarity of miRNA biomarkers.

## Supplementary Information

Supplementary Information.

## Data Availability

The datasets generated and/or analyzed during the current study are included in this published article and its supplementary information files.
